# A nomogram for predicting the nature of thyroid adenomatoid nodules on ultrasound: a dual-center study

**DOI:** 10.3389/fonc.2025.1549866

**Published:** 2025-05-15

**Authors:** Sheng Cheng, Xian-Tao Zeng, Xia Liang, Zhi-Liang Hong, Jian-Chuan Yang, Zi-Ling You, Song-Song Wu

**Affiliations:** ^1^ Department of Ultrasound, Fuzhou University Affiliated Provincial Hospital, Fujian Provincial Hospital, Shengli Clinical Medical College of Fujian Medical University, Fuzhou, Fujian, China; ^2^ Department of Ultrasound, First Affiliated Hospital of Fujian Medical University, National Regional Medical Center, Fujian Medical University, Fuzhou, Fujian, China

**Keywords:** thyroid neoplasms, radiomics, ultrasonography, machine learning, nomogram

## Abstract

**Purpose:**

Thyroid Imaging Reporting and Data System (TIRADS) does not perform well in thyroid adenomatoid nodules on ultrasound (TANU). Therefore, we aimed to generate and validate a nomogram based on radiomics features and clinical information to predict the nature of TANU.

**Methods:**

A total of 200 TANU in 200 patients were enrolled. Firstly, radiomics nomograms (R_Nomogram) and clinical nomograms (C_Nomogram) were constructed using eight machine-learning algorithms. The best R_Nomogram and C_Nomogram generated the Radiomics-clinical nomogram (R-C_nomogram). We compared the Area under the receiver operating characteristic curve (AUC), calibration curve, and decision curve analysis (DCA) of different nomograms. The unnecessary intervention rates were compared between nomograms and the 2017 ACR TI-RADS recommendations.

**Results:**

The R-C_Nomogram had a higher AUC than other nomograms [training cohort: R-C_Nomogram (AUC: 0.922) Vs. C_Nomogram (AUC: 0.825): *p*<0.001, R-C_Nomogram Vs. R_ Nomogram (AUC:0.836), *p*=0.007); validation cohort: R-C_Nomogram (AUC: 0.868) Vs. C_Nomogram (AUC: 0.850): *p*=0.778, R-C_Nomogram Vs. R_Nomogram (AUC:0.684), *p*=0.005). The R-C_Nomogram has the lowest unnecessary intervention rate among all approaches.

**Conclusion:**

The R-C_Nomogram exhibited excellent diagnostic performances for predicting the nature of TANU. By incorporating clinical and radiomics features, the R-C Nomogram can reduce unnecessary biopsies and guide treatment decisions such as ultrasound-guided thermal ablation, improving patient management and reducing healthcare resource burden.

## 1 Introduction

Follicular thyroid neoplasm (FTN) includes follicular thyroid adenoma (FTA), follicular thyroid carcinoma (FTC), follicular variant papillary thyroid carcinoma (FVPTC), borderline follicular tumors, and so on (1). FTA’s typical ultrasound (US) findings are homogeneous isoechoic or hypoechoic nodules parallel to the skin surface with well-defined margins and peripheral halo without calcifications and abnormal lymph node enlargement. Those US findings occasionally also appear on FTC, papillary thyroid carcinoma (PTC), medullary carcinoma (MC), and other malignant thyroid tumors (2). Therefore, in this study, those US findings referred to FTA were defined as thyroid adenomatoid nodules on US (TANU). There has yet to be a consensus on the recommendations about TANU in Thyroid Imaging Reporting and Data Systems (TIRADS).

Most TANUs have long-term durations and big sizes since their benign appearance; fine-needle aspiration (FNA) is often performed. However, it may cause many unnecessary FNA because the malignancy risk of TANU varies from 0% to 25.4% in different TIRADS (3–6). In addition, most results of TANUs’ FNA are Bethesda category IV, which is not determinative of malignancy or benign, and most surgical pathology results of Bethesda category IV are benign (7). Further methods, such as ultrasound elastography, core needle biopsy (CNB), and molecular testing, need to be revised to accurately determine the nature of TANU.

The American College of Radiology Thyroid Imaging Reporting and Data System (ACR-TIRADS), introduced in 2017 (2017 ACR-TIRADS), however, reveals critical shortcomings in the recommendation of TANU. While TR3 nodules show robust NPV (94.6% against cytology, 100% against histopathology), higher-risk categories underperform. TR4 nodules exhibit a mere 6.1% PPV for malignancy histologically, rising to 66.7% for TR5. This gradient fails to align with clinical urgency, as 10.9% of resected TR4 nodules proved malignant despite lower scores. Size-based exacerbate this issue: 30.7% of small (<1.5 cm) TR4 and 50% of TR5 nodules omitted from FNAC per guidelines harbored malignancies (8). This suggests that the system may overestimate the risk of malignancy in these nodules, leading to unnecessary biopsies and patient anxiety, especially in resource-limited countries like China and India. Preoperatively and accurately diagnosing the nature of TANU will also facilitate the use of ultrasound-guided thermal ablation, which has proven effective for benign nodules (9, 10). Therefore, there is an urgent clinical need for a more effective diagnostic method to determine the nature of TANU preoperatively.

Radiomics, the extraction of quantitative features from medical images, has emerged as a promising tool in evaluating and managing thyroid carcinoma. Integrating radiomics with advanced imaging techniques enhances the ability to differentiate between benign and malignant thyroid nodules, predict treatment outcomes, and assess disease progression. Integrating machine learning (ML) algorithms with radiomic data enhances predictive modeling capabilities. By analyzing large datasets of extracted features, ML models can identify patterns associated with malignancy or treatment response that may not be apparent through visual assessment alone (11–14).

Clinical information, such as age and gender, are also related to the prognosis and risk of thyroid carcinoma. Younger patients generally have better survival outcomes compared to older individuals, reinforcing the importance of age as a critical factor in both the diagnosis and prognosis of thyroid cancer (15). Thyroid carcinoma is diagnosed approximately 2.9 to 4 times more often in women than in men; this disparity is especially pronounced in the case of PTC (16). However, the role of age and gender in predicting the nature of TANU is still unknown.

To date, there is no study about whether integrating ML algorithms, radiomics features, and clinical information can help predict the nature of TANU and reduce unnecessary biopsies. Therefore, this study aims to generate and validate a nomogram that integrates radiomics features and clinical information using ML algorithms to predict the nature of TANU and compare the unnecessary biopsy rates between the nomogram and the 2017 ACR-TIRADS.

## 2 Materials and methods

### 2.1 Patients

**Figure 1:**
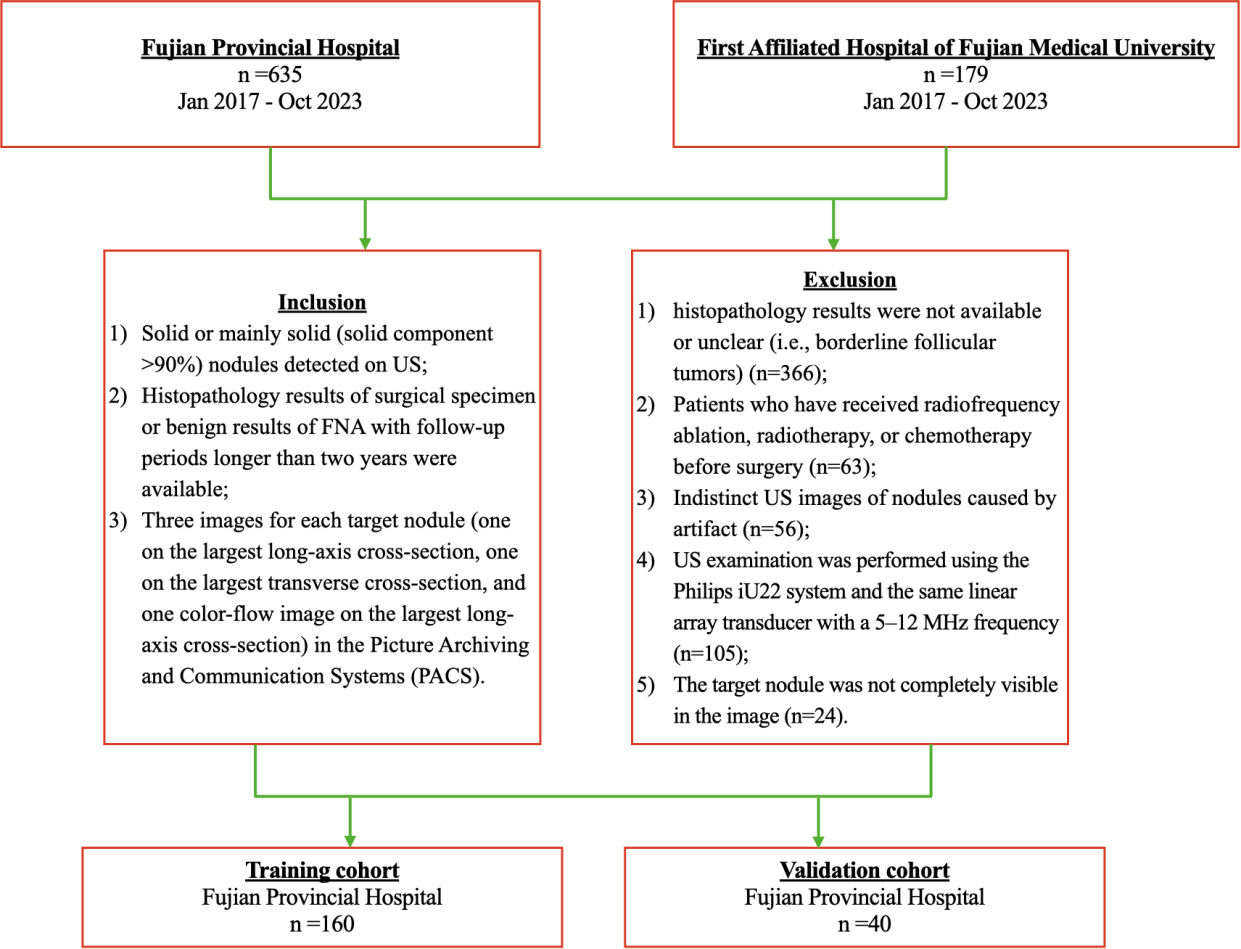
Flowchart of the study population.

Ethical approval was obtained for this retrospective study in our institution, and the informed consent requirement was waived for this retrospective study (K2025-02-182). From January 2017 to October 2023, 852 consecutive patients with thyroid solid nodules at Fuzhou University Affiliated Provincial Hospital (Hospital 1, n=635) and First Affiliated Hospital of Fujian Medical University (Hospital 2, n=217) were included. The patient recruitment pathway is shown in **Figure 1**. Two radiologists with 3 and 10 years of experience, respectively, strictly performed the following inclusion and exclusion criteria.

Inclusion criteria: (1) Solid or mainly solid (solid component ≥90%) nodules that are hyperechoic, isoechoic, or hypoechoic; (2) Histopathology results of the surgical specimen or benign results of FNA with follow-up periods longer than two years were available; (3) Three images for each target nodule (one on the largest long-axis cross-section, one on the largest transverse cross-section, and one color-flow image on the most significant long-axis cross-section) in the Picture Archiving and Communication Systems (PACS).

Exclusion criteria: (1) Distinctly hypoechoic Nodules. (2) Patients who have received radiofrequency ablation, radiotherapy, or chemotherapy before surgery; (3) Indistinct US images of nodules caused by artifact. (4) Histopathology results were not available or unclear (i.e., borderline follicular tumors); (5) The target nodule was not completely visible in the image.

Finally, 160 TANU in 160 patients [49 males and 111 females; 45.77± 12.91 years; range, 15 to 74 years] from Hospital 1 were enrolled as the training cohort. A total of 40 TANU in 40 patients [13 males and 27 females; 49.25± 13.11 years; range, 17 to 81 years] from Hospital 2 were enrolled as the validation cohort. **Supplementary Figure 1** shows different kinds of TANU and their corresponding surgical pathological outcomes. All surgeries were performed for curative intent. Baseline clinicopathologic data, including age and gender, were derived from medical records, and dates of ultrasound examination were also recorded.

### 2.2 Ultrasound image acquisition

US examinations used the Philips iU22 system and linear array transducer (L12-5; Philips Ultrasound, Bothell, Washington). The radiologist who performed the US examination adjusted the imaging parameters and acquired the images. Three images were routinely recorded for each target nodule (one on the largest transverse cross-section, one on the largest long-axis cross-section, and one color-flow image on the largest long-axis cross-section). The radiologist also acquired more images containing important features (calcification, halo, extrathyroidal extension, etc.). All images were stored in the PACS. A radiologist with over three years of experience in thyroid ultrasound using ITK-SNAP 3.8.0 (http://www.itksnap.org) to draw an outline of the region of interest (ROI), which was the target region for radiomics feature extraction (**Supplementary Figure 2**).

### 2.3 Extraction of radiomics features

The feature extraction task was completed using Python 3.11.5 in the training cohort. The handcrafted features can be divided into three groups: (I) geometry, (II) intensity, and (III) texture. The geometry features describe the three-dimensional shape characteristics of the tumor. The intensity features describe the first-order statistical distribution of the voxel intensities within the tumor. The texture features describe the patterns or the intensities’ second and high-order spatial distributions. Here, the texture features are extracted using several different methods, including the gray-level co-occurrence matrix (GLCM), gray-level run length matrix (GLRLM), gray-level size zone matrix (GLSZM), and neighborhood gray-tone difference matrix (NGTDM) methods. Wavelet filtering and Laplacian of Gaussian spatial band-pass filter were used to derive image features at different spatial scales by tuning the filter parameter between 1.0 and 3.0 (1.0, 2.0, 3.0).

### 2.4 Feature selection

Statistics: We also conducted the Mann-Whitney U statistical test and feature screening for all radiomics features. Only the *p*<0.05 of the radiomics feature was kept.

Correlation: For features with high repeatability, Spearman’s rank correlation coefficient was also used to calculate the correlation between features, and one of the features with a correlation coefficient greater than 0.9 between any two features is retained. We use a greedy recursive deletion strategy for feature filtering to maintain the ability to depict features to the greatest extent. That is, the feature with the most excellent redundancy in the current set is deleted each time. After this, 23 features were finally kept.

LASSO: The least absolute shrinkage and selection operator (LASSO) regression model was used on the training data set for signature construction. To find an optimal λ, 10-fold cross-validation with minimum criteria was employed, where the final value of λ yielded minimum cross-validation error. Depending on the regulation weight λ, LASSO shrinks all regression coefficients towards zero and sets the coefficients of many irrelevant features precisely to zero. The retained features with nonzero coefficients were used for regression model fitting and combined into a radiomics signature. Subsequently, we obtained a radiomics score for each patient by a linear combination of retained features weighed by their model coefficients. The Python scikit-learn package was used for LASSO regression modeling.

### 2.5 Radiomics nomogram

After Lasso feature screening, we input the final features into the ML models like logistic regression (LR), support vector machine (SVM), K-nearest Neighbors (KNN), Extra Trees (ET), Random Forests (RF), eXtreme Gradient (XGboost), Light Gradient Boosting Machine (LightGBM), multi-layer perceptron (MLP) for risk model construction. Here, we adopted 5-fold cross-verification to obtain the final radiomics signature. The radiomics signature of the highest AUC of the ML algorithm in the validation cohort was used to construct the radiomics nomogram (R_Nomogram).

### 2.6 Clinical nomogram

The building process of the clinical nomogram (C_Nomogram) was almost the same as the radiomics signature. First, univariable and multivariable analyses selected the features used to build the C_nomogram. We also used the same ML models in the R_Nomogram building process. 5-fold cross-validation and validation cohort were set to be fixed for fair comparison.

### 2.7 Radiomics-clinical nomogram

Radiomics-Clinical Nomogram (R-C_Nomogram) was established in combination with R_Nomogram and C_Nomogram. The diagnostic efficacy of nomograms was tested in training and validation cohorts; ROC curves were drawn to evaluate the diagnostic efficacy of nomograms. The calibration efficiency of nomograms was assessed by drawing calibration curves, and the Hosmer-Lemeshow analytical fit was also used to evaluate the calibration ability of nomograms. Mapping decision curve analysis (DCA) to assess the clinical utility of nomograms.

### 2.8 Recommendations of 2017 ACR TI-RADS

Images analyses were based on the cross-sectional static images of nodules in the PACS rather than real-time US. Two radiologists with ten years of experience assessed all 200 nodules according to the 2017 ACR TI-RADS. Each nodule was scored using five lexicon categories: composition, echogenicity, shape, margin, and echogenic foci. The scores were recorded for each nodule according to these five lexicon categories. The corresponding sums of scores reflect the ACR scores of the nodules. The two radiologists were blinded to the other evaluations and outcomes. Different scores were agreed upon through negotiation between the two radiologists for clarity and analysis. FNA recommendations were according to the criteria of the 2017 ACR TI-RADS and the malignancy prediction of R_C_Nomogram. The unnecessary intervention rates were compared between the R_C_Nomogram and 2017 ACR TI-RADS.

### 2.9 Statistical analysis

The statistical analysis and plots were performed using Python (3.12.4) and R (version 4.2.1). Mean ± standard deviation and median (interquartile range) were used to describe continuous data where appropriate. Categorical variables were reported as number of cases and percentages. Accuracy (ACC), AUC, Sensitivity (SEN), specificity (SPE), positive prediction value (PPV), negative prediction value (NPV), Precision, Recall, and F1 score were used to evaluate different ML models. AUC was used to compare the diagnostic performance between different ML models using the Delong method. A *P*-value less than 0.05 was considered statistically significant.

## 3 Results

### 3.1 Histopathological results and clinical characteristics

Among the 200 TANUs that finally enrolled in this study, 95 TANUs had pre-operative FNA; among them, 35 TANUs without malignant appearances during the two years of follow-up were considered benign. A total of 165 TANUs had surgeries without FNA. **Supplementary Figure 3** illustrates the pathological or follow-up results of all TANUs. The baseline characteristics of patients in cohorts are shown in **Table 1**.

**Table 1:** Baseline characteristics of patients.

Index	Training (benign)	Training (malignancy)	P value	Validation (benign)	Validation (malignancy)	P value
Age	42.67 ± 11.92	48.95 ± 13.18	<0.001	46.40 ± 14.84	52.10 ± 10.74	0.172
Size	32.98 ± 9.92	37.72 ± 13.57	0.013	31.07 ± 11.73	36.00 ± 14.24	0.239
Gender			0.004			0.007
Female	65(80.25)	46(58.23)		18(90.00)	9(45.00)	
Male	16(19.75)	33(41.77)		2(10.00)	11(55.00)	
ACR-TIRADS Scores	3.43 ± 1.04	5.19 ± 1.81	<0.001	3.40 ± 0.75	6.15 ± 1.63	<0.001

ACR-TIRADS, Thyroid Imaging Reporting and Data System of American College of Radiology.

### 3.2 Signature building

Features Statistics: 6 categories and 1555 handcrafted features are extracted, including 305 first-order features, 14 shape features, and the last are texture features. All handcrafted features are extracted with an in-house feature analysis program implemented in Pyradiomics (http://pyradiomics.readthedocs.io). **Supplementary Figure 4** shows all features and corresponding *p*-value results.

### 3.3 LASSO feature selection

**Figure 2:**
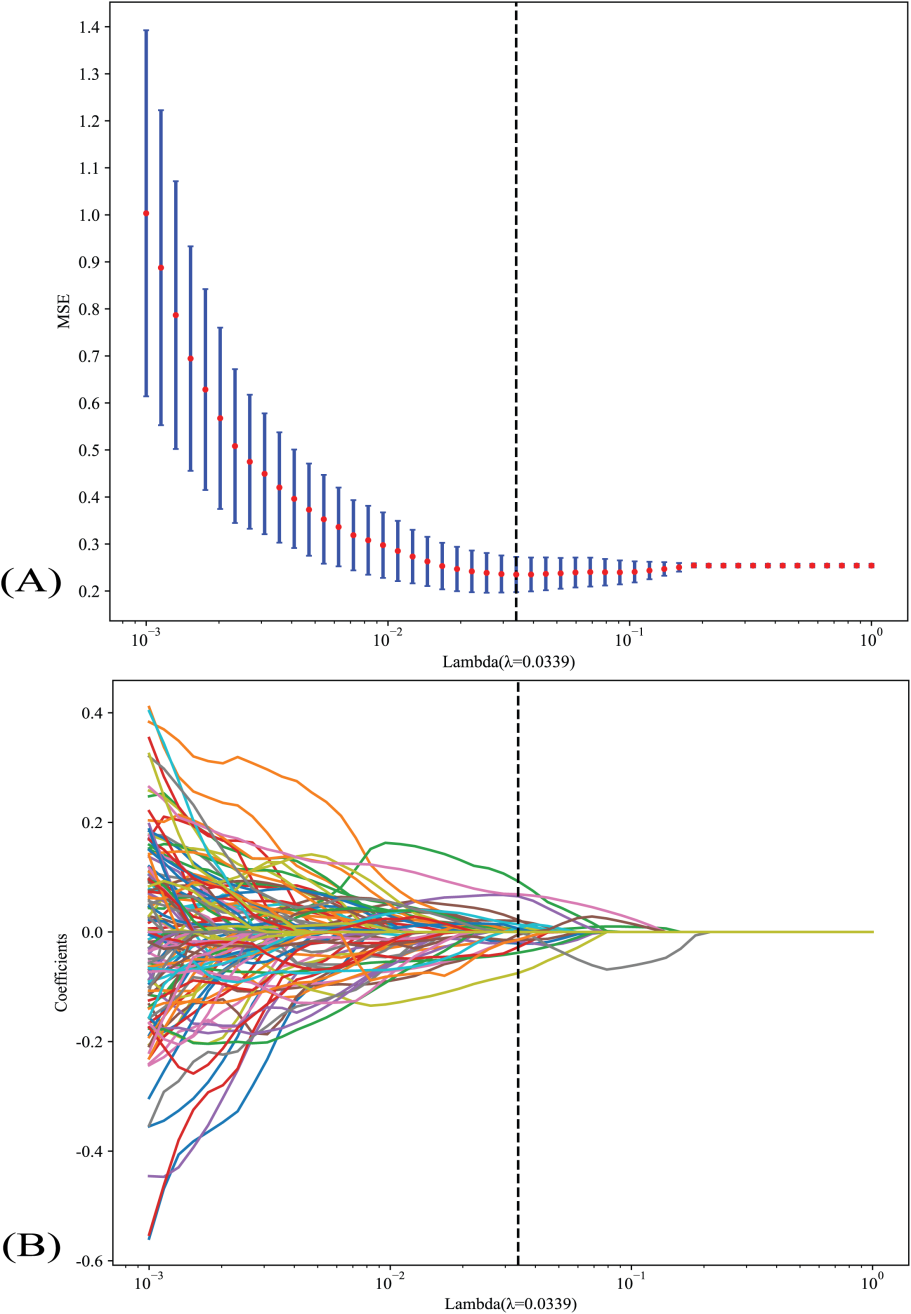
**(A)** Coefficients and MSE (mean standard error) of 10-fold validation; **(B)** LASSO analysis of indicators. LASSO, the least absolute shrinkage and selection operator.

Nonzero coefficients were selected to establish the Rad-score with a least absolute shrinkage and selection operator (LASSO) logistic regression model. Coefficients and MSE (mean standard error) of 10-fold validation are shown in **Figure 2**. The formula for the Rad-score is shown in **Supplementary Table 1**. The coefficient value in the final selected non-zero features is shown in **Supplementary Figure 5**.

### 3.4 Radiomics nomogram

**Supplementary Table 2** shows the metrics of radiomics models we used to predict the nature of TANU. The KNN achieved the best value of AUC in the training and validation cohort, reaching 0.858 and 0.838, respectively. **Supplementary Figure 6A** shows each radiomics model’s AUC on the validation cohort. Therefore, the KNN model was selected as the base model in building the R_Nomogram.

### 3.5 Clinical nomogram

Univariable and multivariable analyses of clinical features were demonstrated in **supplementary Figure 7**. Finally, gender (male) and ACR TI-RADS scores were selected to generate the clinical models. In this study, age is not related to malignancy. **Supplementary Figure 6B** shows each clinical model’s AUC on the validation cohort. The LR model performed the best in the validation cohort (AUC: 0.925). LR model was therefore selected as the base model in the building of the C_Nomogram.

### 3.6 Radiomics-clinical nomogram

**Figure 3:**
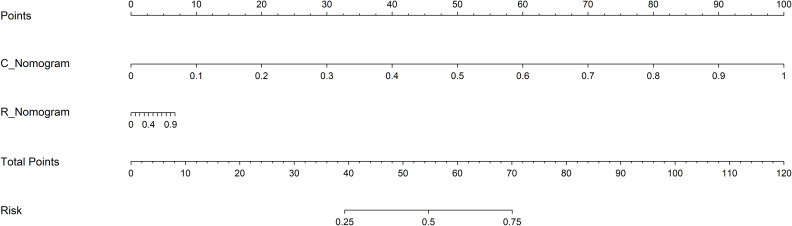
The R-C Nomogram. R_Nomogram, radiomics nomogram; C_Nomogram, clinical nomogram; R-C_Nomogram, radiomics-clinical_Nomogram.

In the training cohort, both R_Nomogram and C_Nomogram get the perfect fitting. In the validation cohort, the R_Nmogram seems overfitting, but the C_Nomogram still fits well. The R-C_Nomogram using the Logistic Regression algorithm was performed to combine the R_Nomogram and C_Nomogram (**Figure 3**).

### 3.7 Comparison of different nomograms

**Figure 4:**
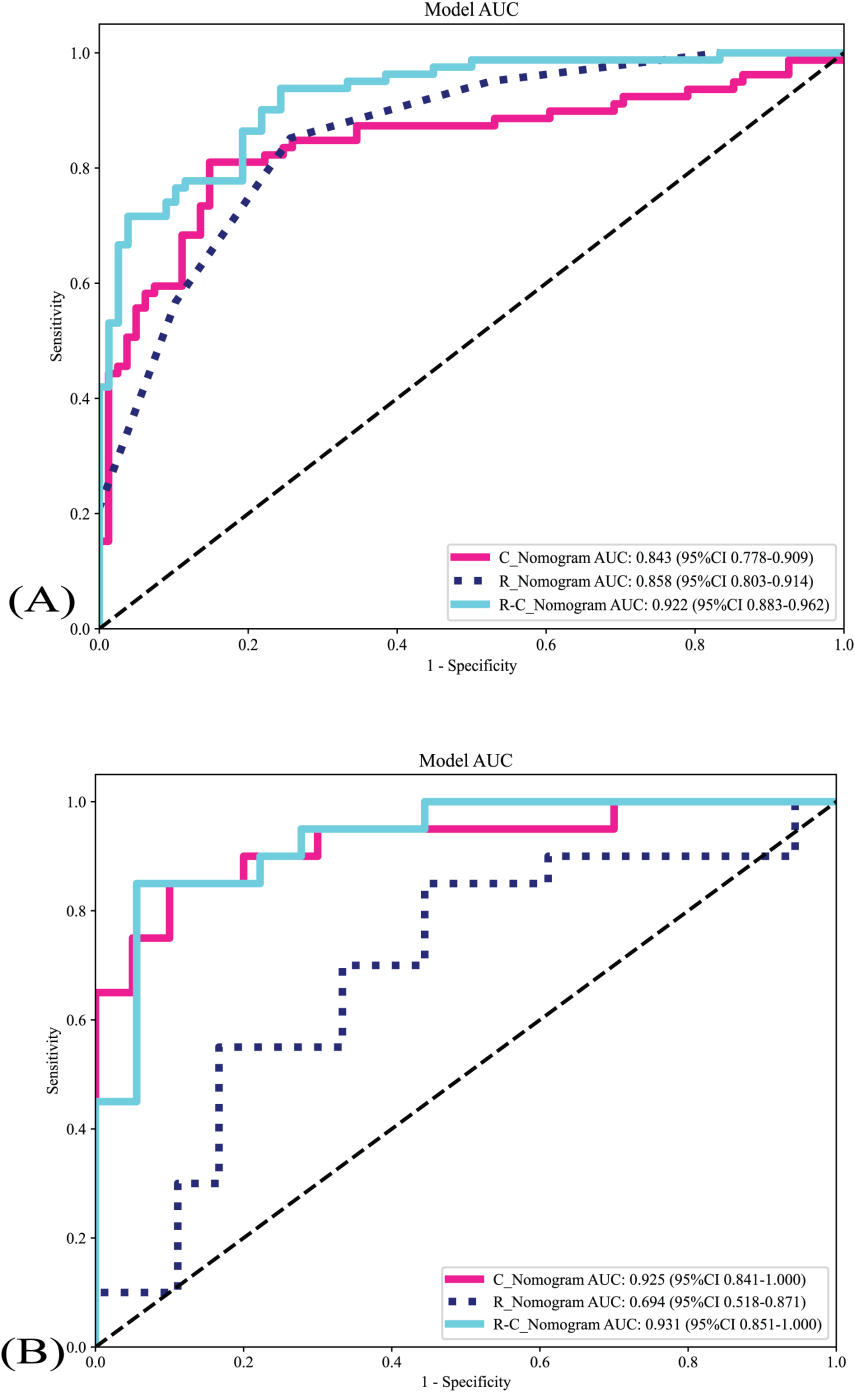
The nomograms’ AUCs. **(A)** The nomograms’s AUCs in the training cohort. **(B)** The nomograms’s AUCs in the validation cohort. R_Nomogram: radiomics nomogram; C_Nomogram: clinical nomogram; R-C_Nomogram: radiomics-clinical_Nomogram.

The metrics of the R_Nomogram, C_Nomogram, and R-C_nomogram are demonstrated in **Table 2**. The nomograms’ AUCs are shown in **Figure 4**. The AUC of the R-C Nomogram (0.931 in the validation cohort) demonstrates excellent predictive accuracy, with high sensitivity (80.0%) and specificity (94.4%), which indicates its potential to guide accurate clinical decisions, such as reducing unnecessary biopsies while identifying high-risk cases for malignancy.

**Table 2:** Metrics of different nomograms for predicting the nature of TANU.

Cohort	Nomogram	ACC	AUC	95% CI	SEN	SPE	PPV	NPV	Precision	Recall	F1	Threshold
Training	C_Nomogram	0.825	0.843	0.778-0.909	0.797	0.852	0.840	0.812	0.840	0.797	0.818	0.481
Training	R_Nomogram	0.730	0.858	0.803- 0.914	0.568	0.897	0.852	0.667	0.852	0.568	0.681	0.600
Training	R-C_Nomogram	0.836	0.922	0.883-0.962	0.914	0.756	0.796	0.894	0.796	0.914	0.851	0.446
Validation	C_Nomogram	0.850	0.925	0.842-1.000	0.800	0.900	0.889	0.818	0.889	0.800	0.842	0.588
Validation	R_Nomogram	0.684	0.694	0.518-0.871	0.800	0.556	0.667	0.714	0.667	0.800	0.727	0.341
Validation	R-C_Nomogram	0.868	0.931	0.851-1.000	0.800	0.944	0.941	0.810	0.941	0.800	0.865	0.629

TANU, Thyroid adenomatoid nodules on ultrasound; ACC, accuracy; AUC, Area under the receiver operating characteristic curve; CI, Confidence Interval; SEN, sensitivity; SPE, specificity; PPV, positive prediction value; NPV, negative prediction value; C_Nomogram, clinical nomogram; R_Nomogram, radiomics nomogram; R-C_Nomogram, radiomics-clinical nomogram.

Delong test was used to compare the AUCs of different nomograms [training cohort: R-C_nomogram *vs*. C_Nomogram (*p*<0.001), R-C_Nomogram *vs*. R_Nomogram (P=0.007); validation cohort: R-C_nomogram *vs*. C_Nomogram (*p*=0.78), R-C_Nomogram *vs*. R_Nomogram (*p*=0.005).

### 3.8 Calibration curves

**Figure 5:**
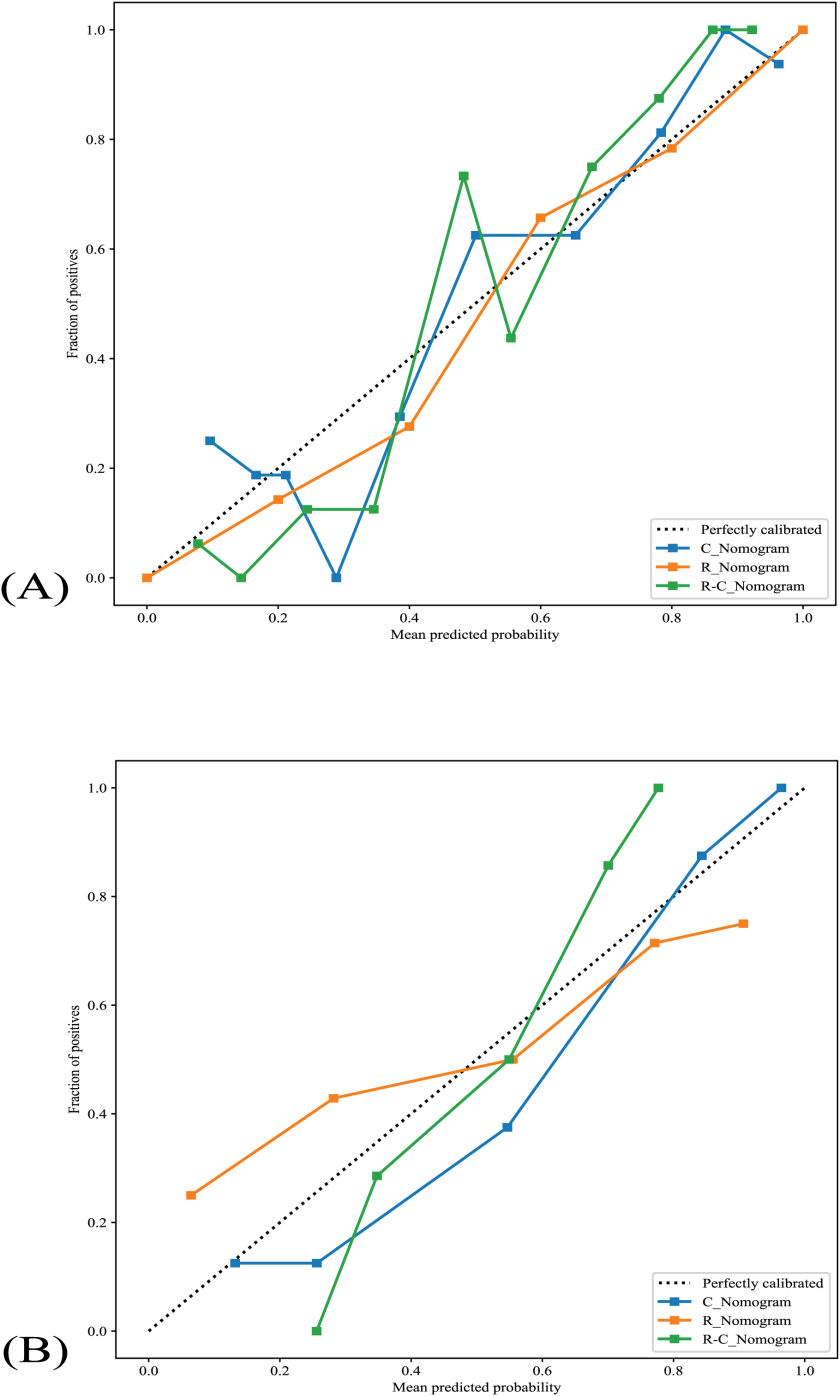
The nomograms’ calibration curves. **(A)** The nomograms’s calibration curves in the training cohort. **(B)** The nomograms’s calibration curves in the validation cohort. R_Nomogram: radiomics nomogram; C_Nomogram: clinical nomogram; R-C_Nomogram: radiomics-clinical_Nomogram.

All nomograms’ calibration curves show good agreement between TANU’s predicted and observed nature in training and validation cohorts (**Figure 5**). The Hosmer-Lemeshow tests [C_Nomogram (*p*=0.181, 0565 for training and validation cohorts, respectively), R_Nomogram (*p*=0.998, 0.050 for training and validation cohorts, respectively), and R-C_nomogram (*p*=0.272, 0.108 for training and validation cohorts, respectively)] indicated that all nomograms fit well in both the training and validation cohorts.

### 3.9 Decision curve analysis

**Figure 6:**
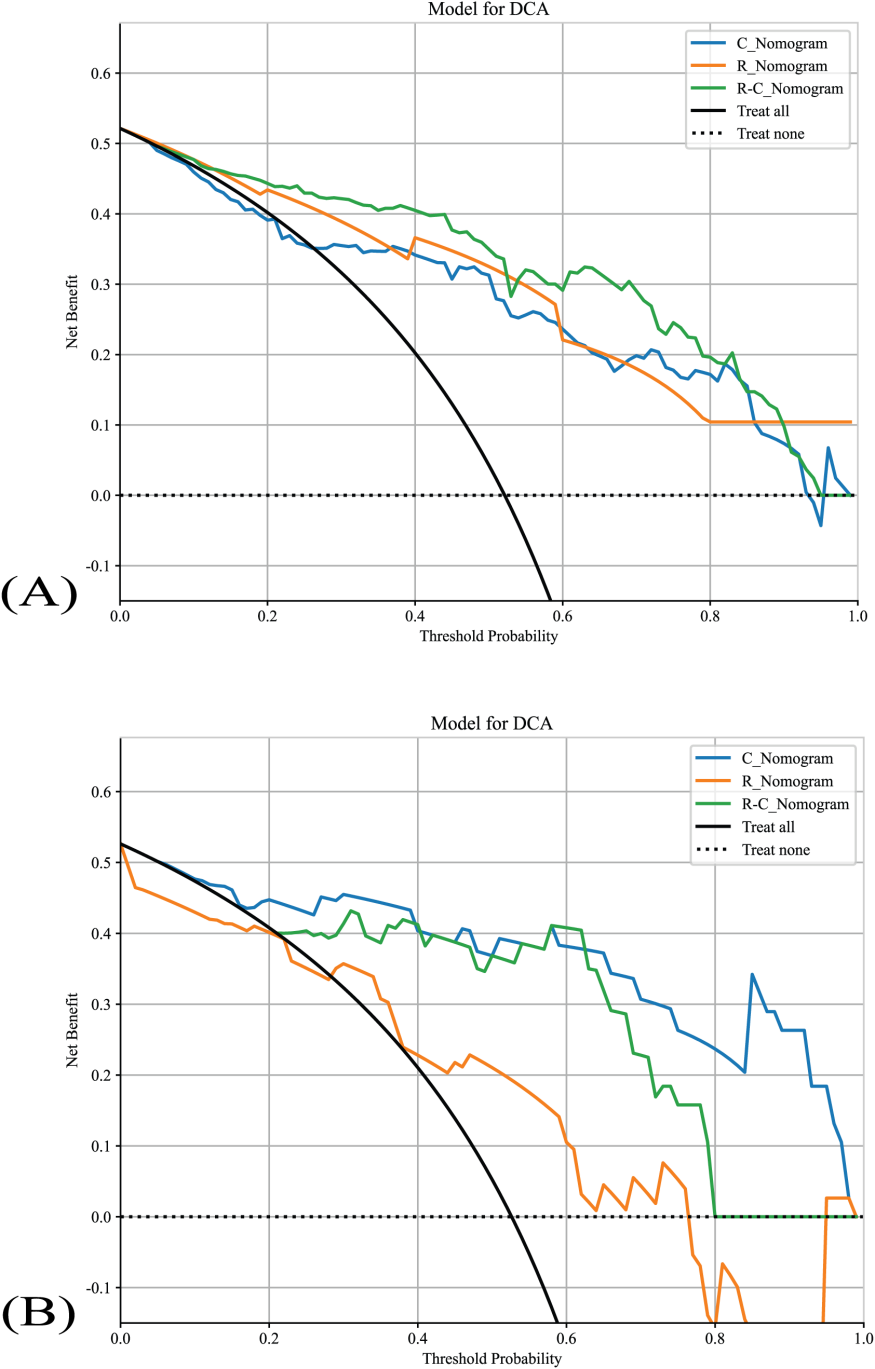
The nomograms’ DCAs. **(A)** The nomograms’s DCAs in the training cohort. **(B)** The nomograms’s DCAs in the validation cohort. R_Nomogram: radiomics nomogram; C_Nomogram: clinical nomogram; R-C_Nomogram: radiomics-clinical_Nomogram.

The DCA of all nomograms is presented in **Figure 6**. Compared with scenarios where no prediction model would be used (i.e., treat-all or treat-none scheme), the R-C_Nomogram showed more benefits for intervention in patients with a prediction probability than other nomograms. Preoperative prediction of TANU using R-C_nomogram has been shown to have better clinical benefit.

### 3.10 Unnecessary intervention rates

The unnecessary intervention rates of all nomograms are shown in **Table 3**. R-C _nomogram’s unnecessary intervention rate is lower than other nomograms and the 2017 ACR-TIRADS.

**Table 3:** Unnecessary intervention rates of different approaches in validation cohort.

Approaches	Recommend intervention	Malignant	Benign	Unnecessary intervention rates
ACR_TIRADS	35	22	13	37.10%
C_Nomogram	21	17	4	19.1%
R_Nomogram	23	17	6	26.1%
R-C_Nomogram	20	17	3	15.0%

ACR, American College of Radiology; TI-RADS, Thyroid Imaging Reporting and Data System; C_Nomogram, clinical nomogram; R_Nomogram, radiomics nomogram; R-C_Nomogram, radiomics combined with clinical nomogram.

## 4 Discussion

The main results of this study are as follows: 1) The R-C_Nomogram performed best in the preoperative prediction of TANU compared to the C_Nomogram, R_Nomogram; 2) The R-C_Nomogram has the lowest unnecessary intervention rate compared to the C_Nomogram, R_Nomogram, and the 2017 ACR-TIRADS.

Prior studies have reported some ML models incorporate US and clinical characteristics to predict thyroid malignancy. Liang et al. developed six ML models incorporating gender, US, strain elastography, and contrast-enhanced US to predict the nature of thyroid nodules. Their results indicated that the LR model had the best diagnostic performance with an AUC of 0.93 (17). Similarly, Luong et al. utilized commonly used ML models to access thyroid nodules with indeterminate diagnoses on FNA and revealed that the RF model performed the best with an AUC of 0.86 (18). Maia et al. developed an LR model based on age, border irregularity, microcalcifications, and nodule size with an accuracy of 81.7% in discriminating malignant from benign thyroid nodules (19). Ouyang et al. observed conventional US features of 1,179 thyroid nodules and found that the RF model achieved the highest AUC of 0.95 (20). Zhang et al. analyzed 2064 thyroid nodules and revealed that ML models, particularly the RF model, diagnose malignant thyroid nodules better than radiologists (21). However, radiomics features are not included in their study.

To date, there is no study about combining ML algorithms, radiomics features, and clinical information to predict the nature of TANU. In terms of radiomics features of the US, Agyekum et al. demonstrated that ML models based on US elastography radiomics features are capable of predicting the likelihood of BRAF^V600E^ mutation in PTC patients with the highest AUC of 0.98 in the SVM_RBF model (22). Li et al. observed extrathyroidal extension (ETE) in children and adolescents with PTC using four ML models based on ultrasound radiomics features. The LightGBM model performed best with an AUC of 0.83 (23).

Regarding clinical factors, some previous studies found that age is not associated with the incidence of thyroid cancer but is related to prognosis (24, 25). Similar to these studies, our data showed that age is not a significant factor in the prediction of TANU. On the other hand, thyroid cancer is predominantly diagnosed in women (26). In contrast, this does not agree with our results that the male was related to malignancy of TANU, although the result was not significant in the multivariable analysis. A prospective study with a larger sample size is needed to validate our results.

Unlike previous ML methodologies that rely solely on radiomic or clinical features, our approach integrates both, leveraging the strengths of each to provide a more accurate, robust prediction of the nature of TANU. This innovative combination improves model performance and helps overcome limitations inherent in using single-source data. In addition, DCA results implied that there would be more benefits to using the R-C_Nomogram for preoperatively predicting the nature of TANU. The disparity was not so significant in the validation cohort, which may be due to the small sample size.

ACR TI-RADS is one of the most used systems. It effectively reduces unnecessary biopsies compared to other TIRADS, which have a 17% to 25% unnecessary intervention rate (27). However, it could perform better for the recommendation of TANU, which is familiar with our data. In contrast, the R-C_Nomogram showed a significantly lower unnecessary intervention rate than the 2017 ACR-TIRADS. This implies that the R-C_Nomogram based on radiomics features and clinical information may be conducive to pre-treatment decision-making in patients with TANU. It can be applied to triage patients requiring FNA in a resource-constrained setting, subsequently guiding appropriate therapy, such as ultrasound-guided thermal ablation.

In a word, the R-C Nomogram offers a promising tool for the preoperative risk stratification of TANU. It could be incorporated into clinical practice through integration into ultrasound reporting software or decision-support systems, assisting clinicians in determining the necessity of biopsy and helping to guide non-invasive management options, such as thermal ablation.

This study has several limitations. First, there is selection bias with a small sample size for the retrospective nature. Second, except for ACR-TIRADS, the diagnostic performances between the ML models and other TIRADS were not analyzed. Despite these limitations, our study has several strengths. It is a dual-center study that includes an external validation cohort, enhancing the generalizability of our results. Furthermore, a comprehensive range of ML models was constructed. Future developments in AI, particularly deep learning models and integrating multi-modal data (e.g., molecular data, histopathological images), hold significant potential to enhance the predictive accuracy of models like the R-C Nomogram, leading to even more personalized treatment recommendations.

## 5 Conclusion

In summary, the R-C_Nomogram exhibited excellent diagnostic performances for predicting the nature of TANU. Using the R-C_nomogram may reduce unnecessary biopsy and facilitate the utility of ultrasound-guided thermal ablation.

## Data Availability

The raw data supporting the conclusions of this article will be made available by the authors, without undue reservation.
