# Supplementary material for: A nomogram for predicting the nature of thyroid adenomatoid nodules on ultrasound: a dual-center study
Source: Front Oncol. 2025 May 15;15:1549866. doi: 10.3389/fonc.2025.1549866 (PMC12119468; doi:10.3389/fonc.2025.1549866)
Supplement: Supplementary file 1 [file Presentation1.pptx]

## Slide 1
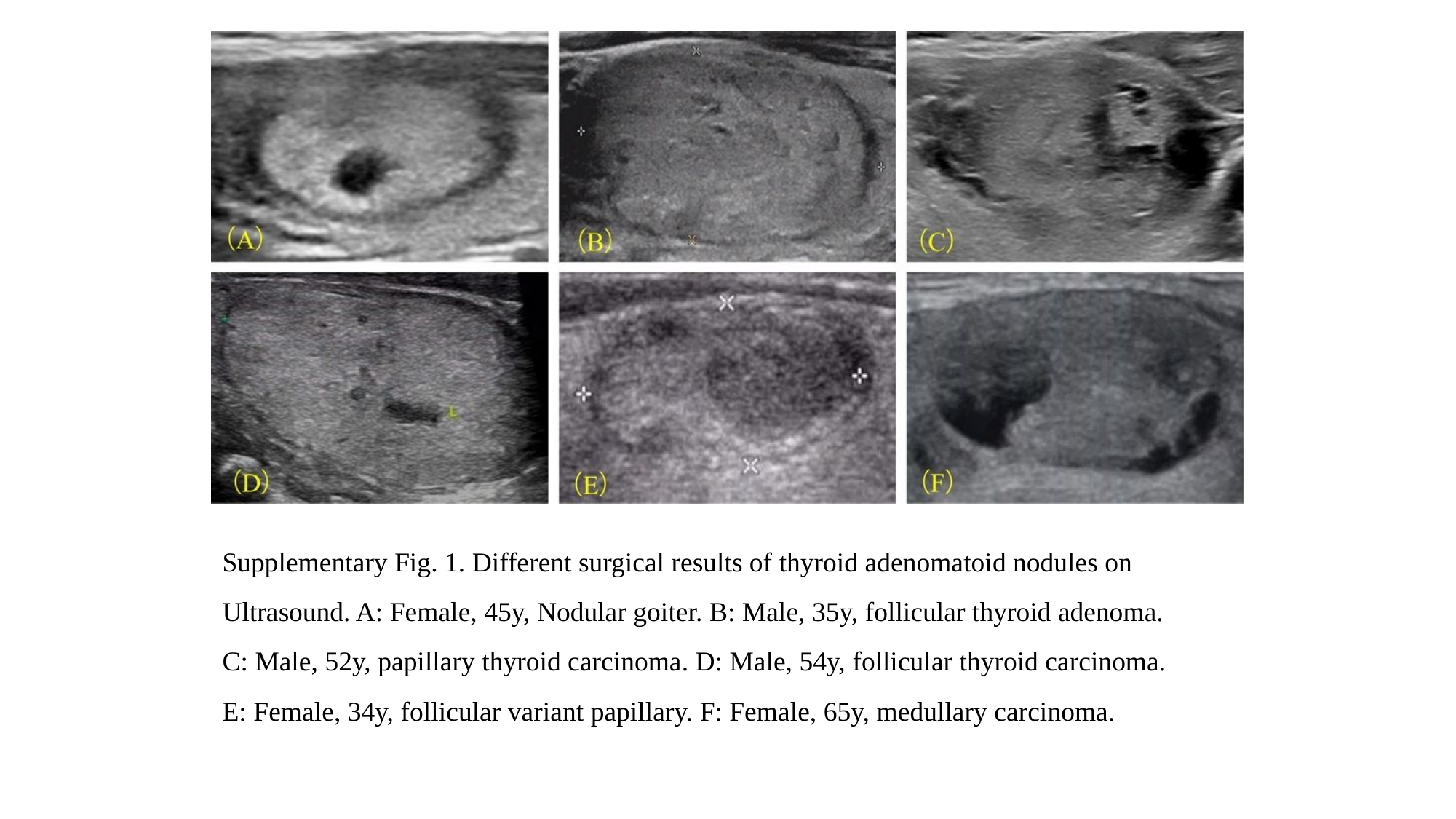

Supplementary Fig. 1. Different surgical results of thyroid adenomatoid nodules on
Ultrasound. A: Female, 45y, Nodular goiter. B: Male, 35y, follicular thyroid adenoma.
C: Male, 52y, papillary thyroid carcinoma. D: Male, 54y, follicular thyroid carcinoma.
E: Female, 34y, follicular variant papillary. F: Female, 65y, medullary carcinoma.

## Slide 2
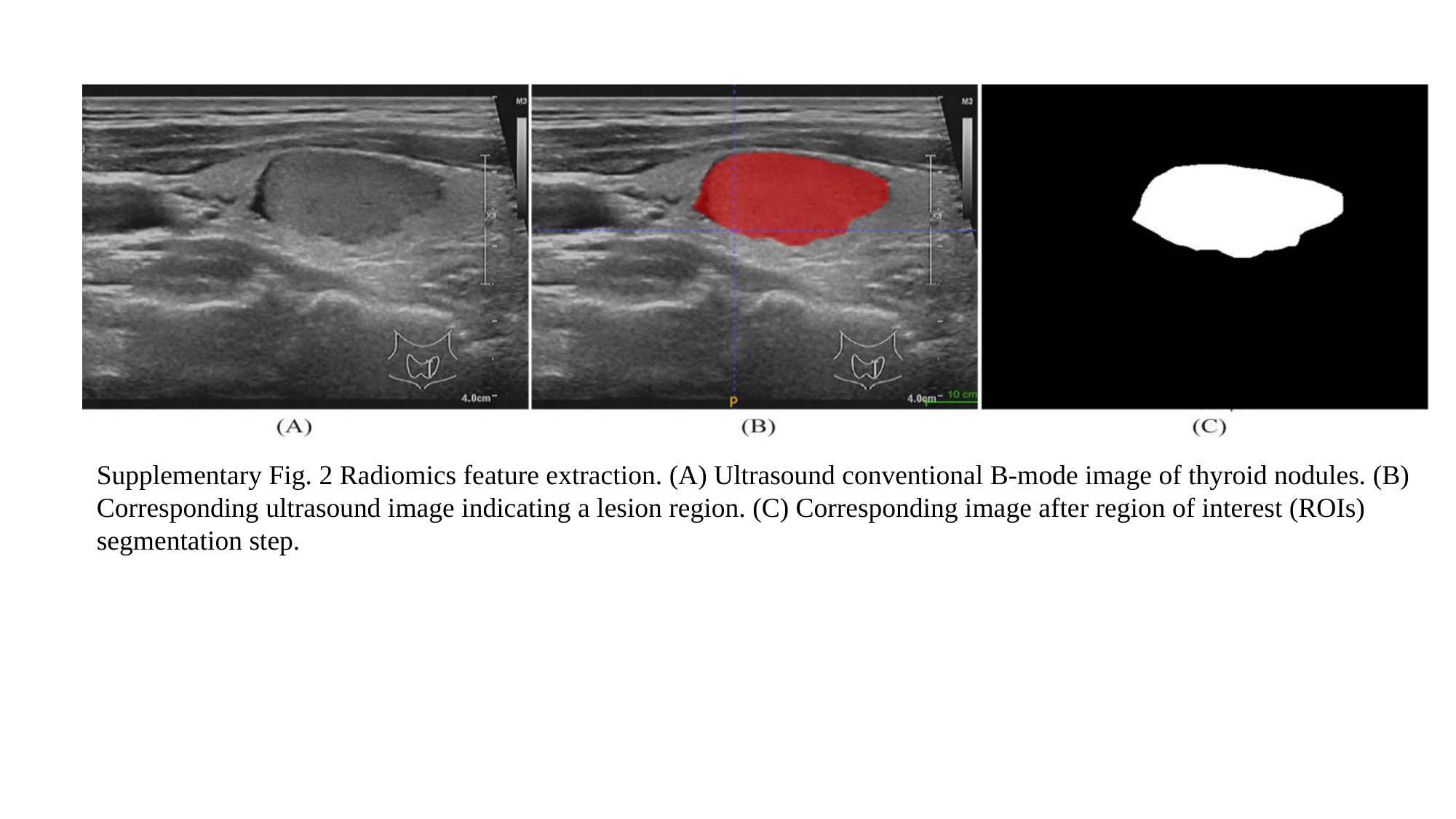

Supplementary Fig. 2 Radiomics feature extraction. (A) Ultrasound conventional B-mode image of thyroid nodules. (B) Corresponding ultrasound image indicating a lesion region. (C) Corresponding image after region of interest (ROIs) segmentation step.

## Slide 3
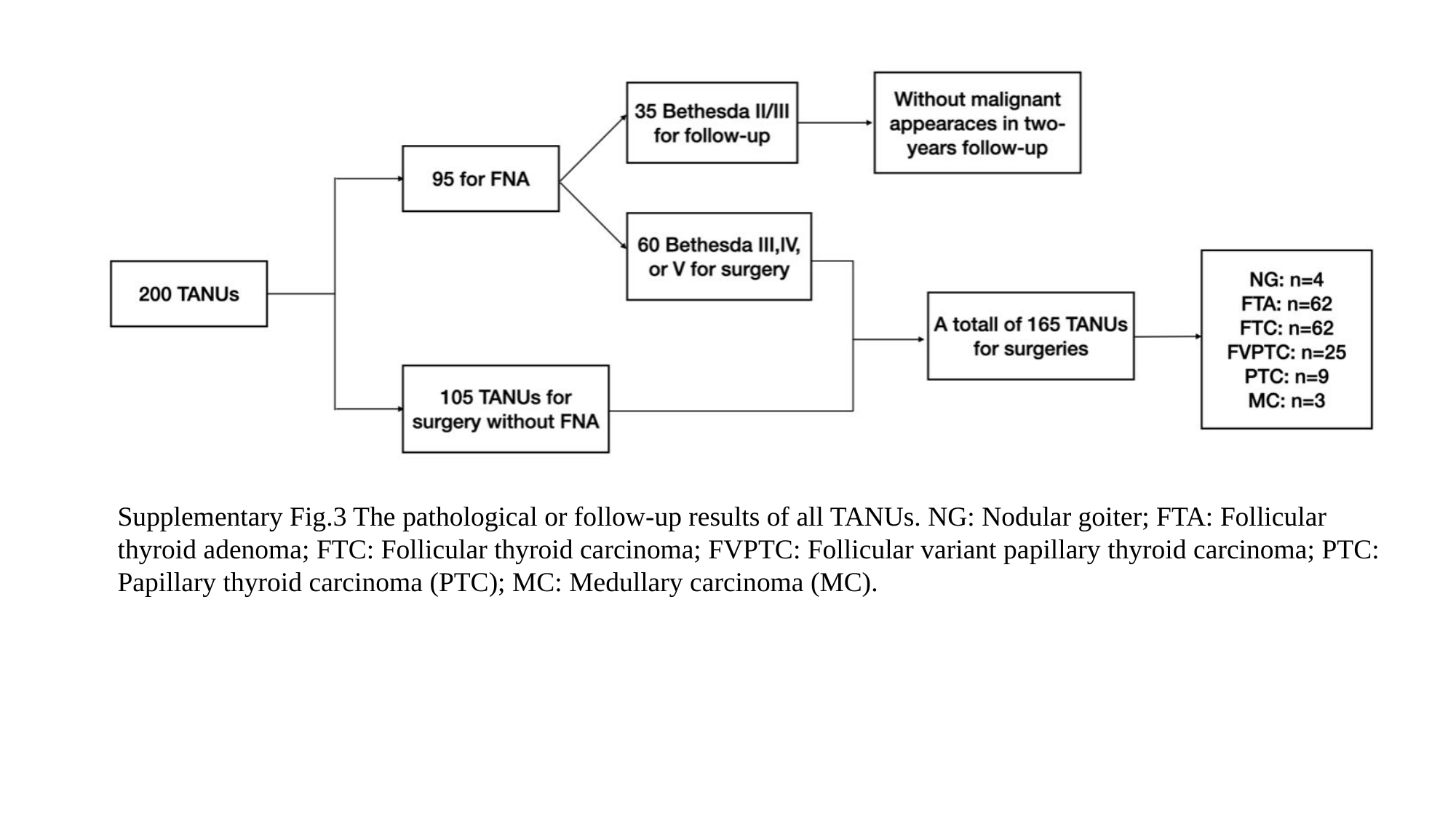

Supplementary Fig.3 The pathological or follow-up results of all TANUs. NG: Nodular goiter; FTA: Follicular thyroid adenoma; FTC: Follicular thyroid carcinoma; FVPTC: Follicular variant papillary thyroid carcinoma; PTC: Papillary thyroid carcinoma (PTC); MC: Medullary carcinoma (MC).

## Slide 4
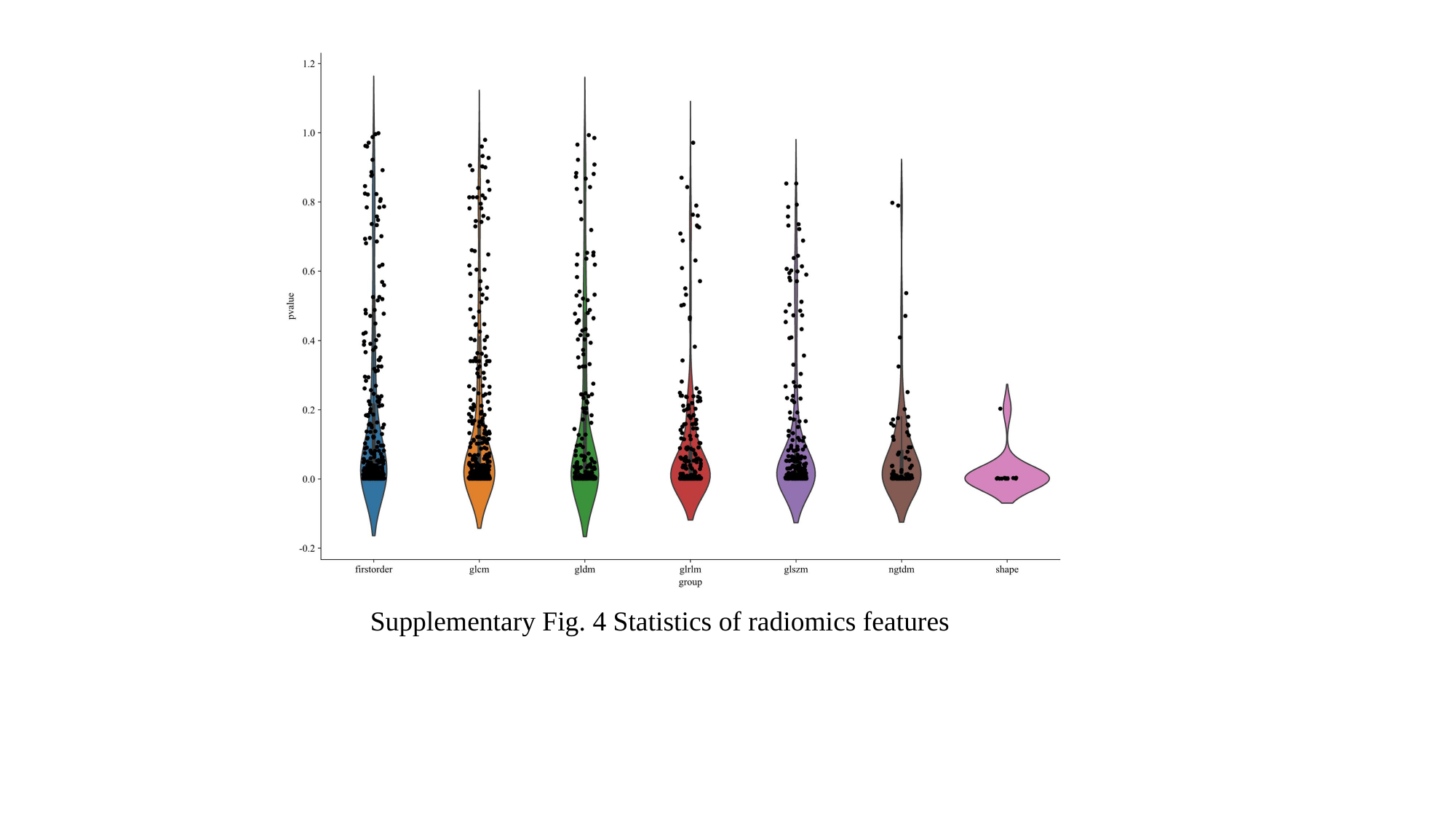

Supplementary Fig. 4 Statistics of radiomics features

## Slide 5
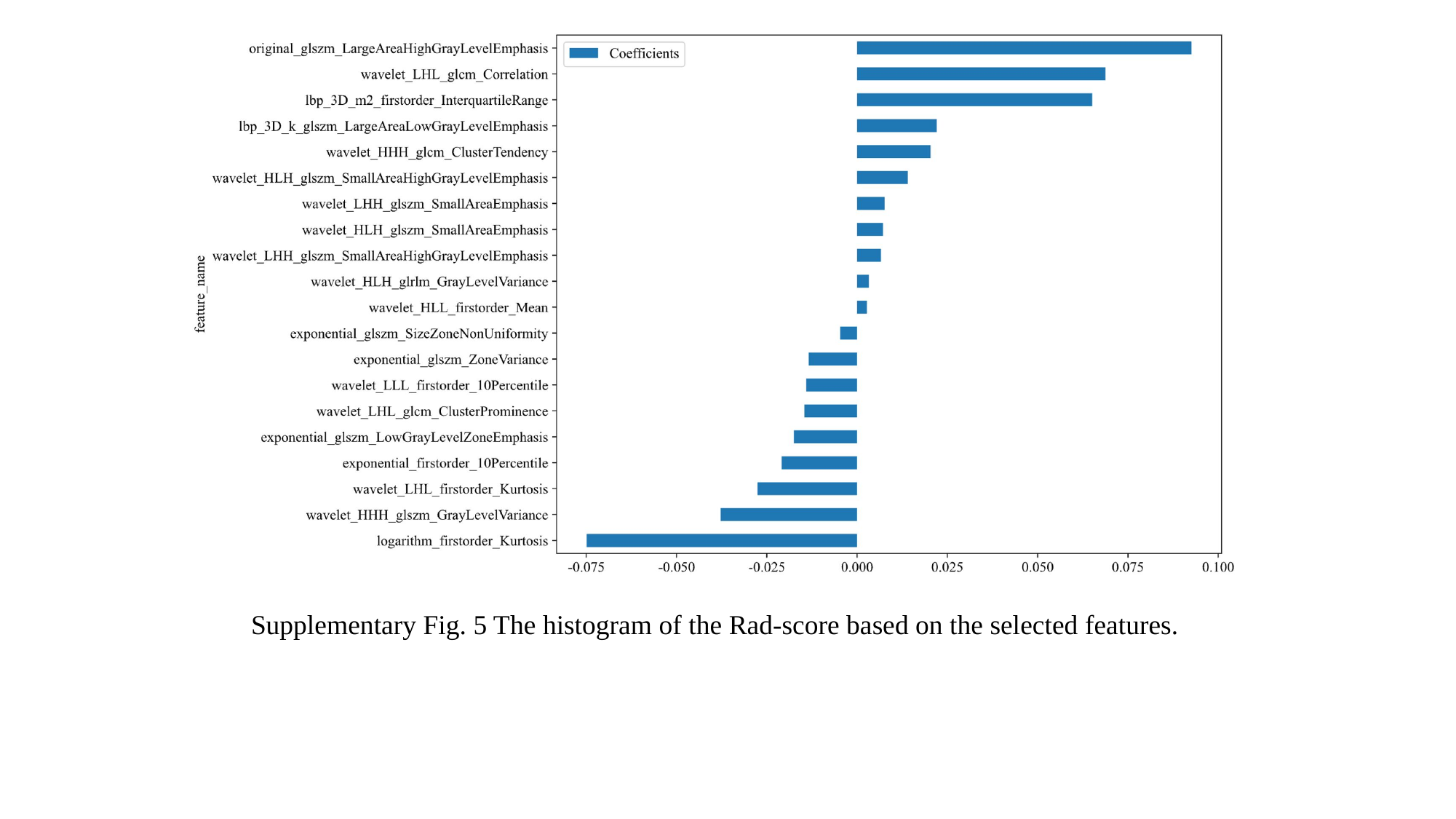

Supplementary Fig. 5 The histogram of the Rad-score based on the selected features.

## Slide 6
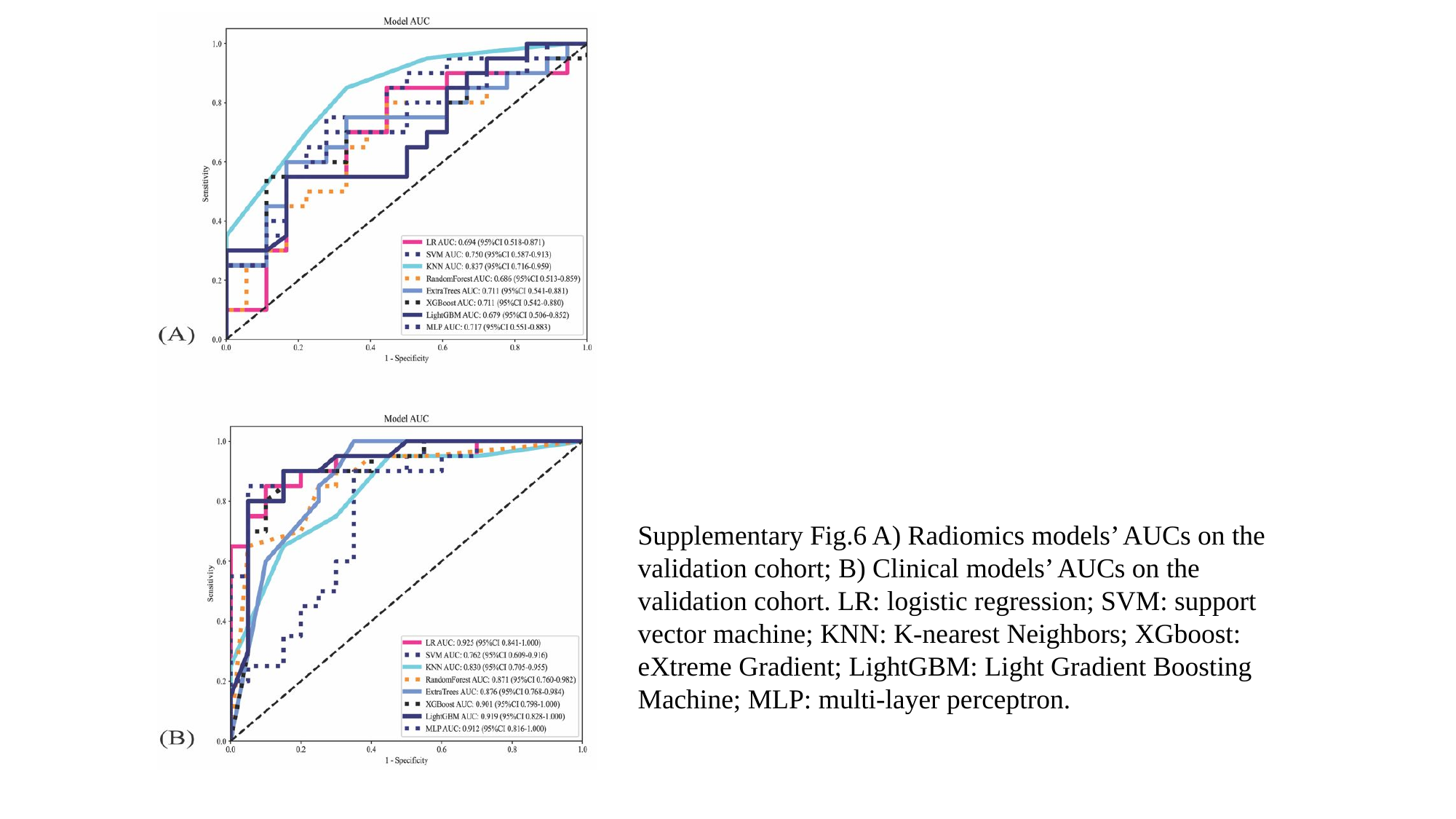

Supplementary Fig.6 A) Radiomics models’ AUCs on the validation cohort; B) Clinical models’ AUCs on the validation cohort. LR: logistic regression; SVM: support vector machine; KNN: K-nearest Neighbors; XGboost: eXtreme Gradient; LightGBM: Light Gradient Boosting Machine; MLP: multi-layer perceptron.

## Slide 7
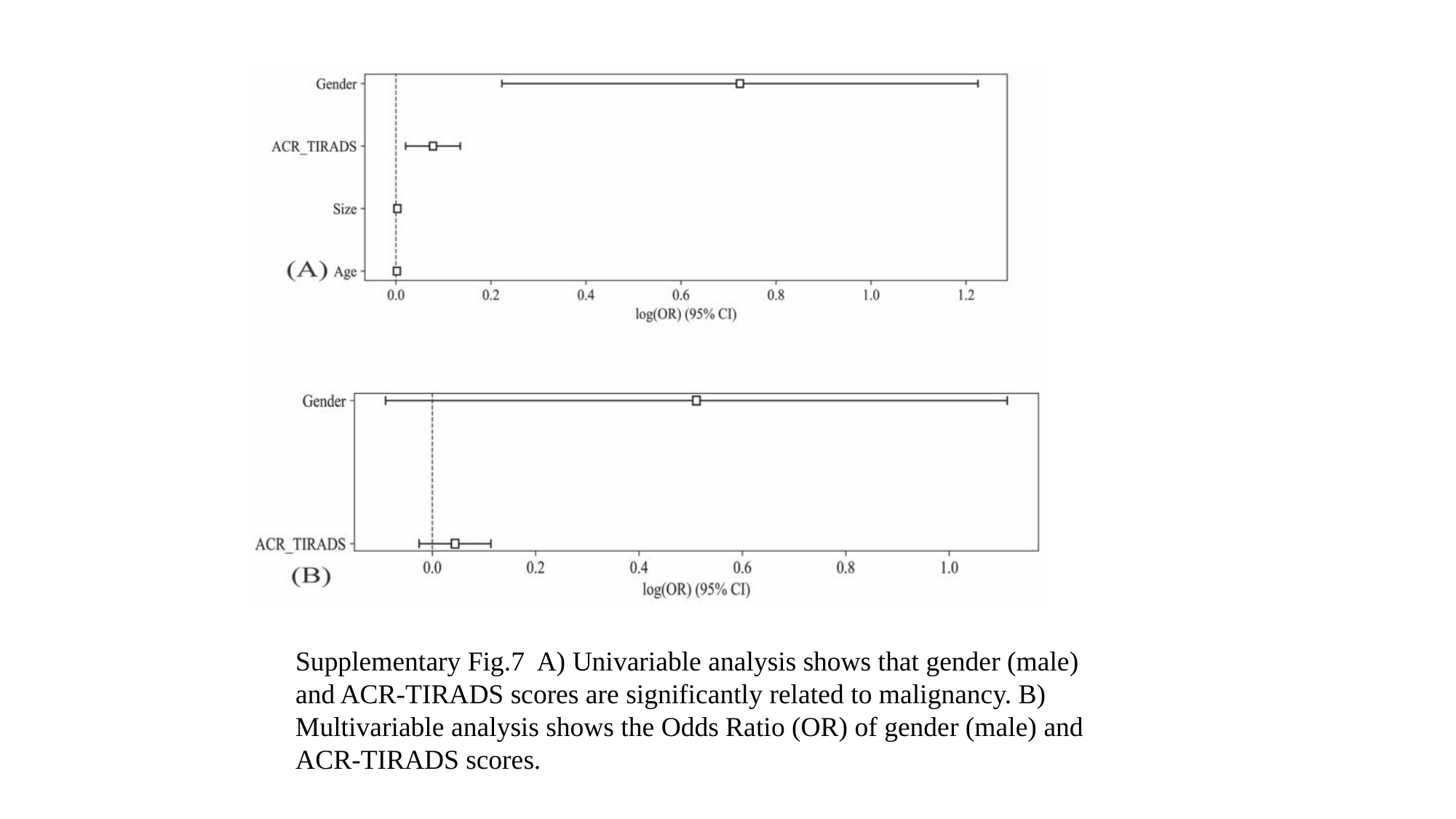

Supplementary Fig.7 A) Univariable analysis shows that gender (male) and ACR-TIRADS scores are significantly related to malignancy. B) Multivariable analysis shows the Odds Ratio (OR) of gender (male) and ACR-TIRADS scores.
