# Supplementary material for: A nomogram for predicting the nature of thyroid adenomatoid nodules on ultrasound: a dual-center study
Source: Front Oncol. 2025 May 15;15:1549866. doi: 10.3389/fonc.2025.1549866 (PMC12119468; doi:10.3389/fonc.2025.1549866)
Supplement: Supplementary file 2 [file Table1.docx]

| Supplementary Table 1 The Coefiicients of Selected Features | |
| --- | --- |
| Feature Name | Coefficient |
| exponential_firstorder_10Percentile | -0.020881 |
| exponential_glszm_LowGrayLevelZoneEmphasis | -0.017521 |
| exponential_glszm_SizeZoneNonUniformity | -0.004709 |
| exponential_glszm_ZoneVariance | -0.013448 |
| lbp_3D_k_glszm_LargeAreaLowGrayLevelEmphasis | +0.022041 |
| lbp_3D_m2_firstorder_InterquartileRange | +0.065075 |
| logarithm_firstorder_Kurtosis | -0.074867 |
| original_glszm_LargeAreaHighGrayLevelEmphasis | +0.092568 |
| wavelet_HHH_glcm_ClusterTendency | +0.020323 |
| wavelet_HHH_glszm_GrayLevelVariance | -0.037809 |
| wavelet_HLH_glrlm_GrayLevelVariance | +0.003237 |
| wavelet_HLH_glszm_SmallAreaEmphasis | +0.007212 |
| wavelet_HLH_glszm_SmallAreaHighGrayLevelEmphasis | +0.014025 |
| wavelet_HLL_firstorder_Mean | +0.002726 |
| wavelet_LHH_glszm_SmallAreaEmphasis | +0.007618 |
| wavelet_LHH_glszm_SmallAreaHighGrayLevelEmphasis | +0.006603 |
| wavelet_LHL_firstorder_Kurtosis | -0.027568 |
| wavelet_LHL_glcm_ClusterProminence | -0.014583 |
| wavelet_LHL_glcm_Correlation | +0.068756 |
| wavelet_LLL_firstorder_10Percentile | -0.014122 |
| Radiomics score= 0.5094339622641509+Features*Coefficients | |
