# Supplementary material for: A nomogram for predicting the nature of thyroid adenomatoid nodules on ultrasound: a dual-center study
Source: Front Oncol. 2025 May 15;15:1549866. doi: 10.3389/fonc.2025.1549866 (PMC12119468; doi:10.3389/fonc.2025.1549866)
Supplement: Supplementary file 3 [file Table2.docx]

| **Supplementary Table 2 Metrics of all radiomics models for predicting the nature of TANU** | | | | | | | | | | | | |
| --- | --- | --- | --- | --- | --- | --- | --- | --- | --- | --- | --- | --- |
| **model_name** | **Accuracy** | **AUC** | **95% CI** | **Sensitivity** | **Specificity** | **PPV** | **NPV** | **Precision** | **Recall** | **F1** | **Threshold** | **Cohort** |
| LR | 0.805 | 0.889 | 0.839-.939 | 0.704 | 0.910 | 0.891 | 0.747 | 0.891 | 0.704 | 0.786 | 0.649 | Training |
| LR | 0.684 | 0.694 | 0.518-0.871 | 0.800 | 0.556 | 0.667 | 0.714 | 0.667 | 0.800 | 0.727 | 0.341 | Validatioin |
| SVM | 0.811 | 0.904 | 0.859- 0.949 | 0.704 | 0.923 | 0.905 | 0.750 | 0.905 | 0.704 | 0.792 | 0.638 | Training |
| SVM | 0.711 | 0.750 | 0.587-0.913 | 0.700 | 0.722 | 0.737 | 0.684 | 0.737 | 0.700 | 0.718 | 0.456 | Validatioin |
| KNN | 0.730 | 0.858 | 0.803- 0.914 | 0.568 | 0.897 | 0.852 | 0.667 | 0.852 | 0.568 | 0.681 | 0.600 | Training |
| KNN | 0.737 | 0.838 | 0.716- 0.959 | 0.700 | 0.778 | 0.778 | 0.700 | 0.778 | 0.700 | 0.737 | 0.600 | Validatioin |
| RandomForest | 0.818 | 0.892 | 0.843-0.940 | 0.765 | 0.872 | 0.861 | 0.782 | 0.861 | 0.765 | 0.810 | 0.570 | Training |
| RandomForest | 0.658 | 0.686 | 0.5135 - 0.8588 | 0.750 | 0.556 | 0.652 | 0.667 | 0.652 | 0.750 | 0.698 | 0.473 | Validatioin |
| ExtraTrees | 0.830 | 0.881 | 0.8266 - 0.9356 | 0.765 | 0.897 | 0.886 | 0.787 | 0.886 | 0.765 | 0.821 | 0.540 | Training |
| ExtraTrees | 0.684 | 0.711 | 0.542 - 0.881 | 0.550 | 0.833 | 0.786 | 0.625 | 0.786 | 0.550 | 0.647 | 0.537 | Validatioin |
| XGBoost | 0.956 | 0.991 | 0.982 - 1.0000 | 0.963 | 0.949 | 0.951 | 0.961 | 0.951 | 0.963 | 0.957 | 0.457 | Training |
| XGBoost | 0.684 | 0.711 | 0.542- 0.880 | 0.500 | 0.889 | 0.833 | 0.615 | 0.833 | 0.500 | 0.625 | 0.590 | Validatioin |
| LightGBM | 0.836 | 0.910 | 0.867-0.953 | 0.778 | 0.897 | 0.887 | 0.795 | 0.887 | 0.778 | 0.829 | 0.534 | Training |
| LightGBM | 0.658 | 0.679 | 0.506-0.852 | 0.500 | 0.833 | 0.769 | 0.600 | 0.769 | 0.500 | 0.606 | 0.581 | Validatioin |
| MLP | 0.761 | 0.844 | 0.786- 0.903 | 0.827 | 0.692 | 0.736 | 0.794 | 0.736 | 0.827 | 0.779 | 0.501 | Training |
| MLP | 0.684 | 0.717 | 0.551-0.883 | 0.650 | 0.722 | 0.722 | 0.650 | 0.722 | 0.650 | 0.684 | 0.532 | Validatioin |
| TANU: Thyroid adenomatoid nodules on ultrasound; LR: logistic regression; SVM: support vector machine; KNN: K-nearest Neighbors; XGboost: eXtreme Gradient; LightGBM: Light Gradient Boosting Machine; MLP: multi-layer perceptron; AUC: Area under the receiver operating characteristic curve; CI: Confidence Interval; PPV: positive prediction value; NPV: negative prediction value | | | | | | | | | | | | |
